# Identification of the regions involved in phonological assembly using a novel paradigm

**DOI:** 10.1016/j.bandl.2015.07.013

**Published:** 2015-11

**Authors:** Tae Twomey, Dafydd Waters, Cathy J. Price, Ferath Kherif, Bencie Woll, Mairéad MacSweeney

**Affiliations:** a ESRC Deafness, Cognition and Language Research Centre, University College London, UK; b Institute of Cognitive Neuroscience, University College London, UK; c Wellcome Trust Centre for Neuroimaging, Institute of Neurology, University College London, UK; d LREN, Department of Clinical Neurosciences, CHUV, University of Lausanne, Switzerland

**Keywords:** fMRI, Reading, Phonological assembly, Phonological memory, Lexical decision, Pars opercularis, Supramarginal gyrus, Precentral gyrus, Pars triangularis

## Abstract

Here we adopt a novel strategy to investigate phonological assembly. Participants performed a visual lexical decision task in English in which the letters in words and letterstrings were delivered either sequentially (promoting phonological assembly) or simultaneously (not promoting phonological assembly). A region of interest analysis confirmed that regions previously associated with phonological assembly, in studies contrasting different word types (e.g. words versus pseudowords), were also identified using our novel task that controls for a number of confounding variables. Specifically, the left pars opercularis, the superior part of the ventral precentral gyrus and the supramarginal gyrus were all recruited more during sequential delivery than simultaneous delivery, even when various psycholinguistic characteristics of the stimuli were controlled. This suggests that sequential delivery of orthographic stimuli is a useful tool to explore how readers, with various levels of proficiency, use sublexical phonological processing during visual word recognition.

## Highlights

• Sequential delivery of letters in words encourages the use of phonological assembly.
• Greater activation in left SMG, POp and precentral gyrus during sequential delivery.
• Activation for ‘phonological assembly’ not confounded with stimulus properties.
• Activation for ‘phonological assembly’ not wholly attributable to processing load.

## 1 Introduction

The process of translating orthography into phonology during reading can occur at multiple levels. These levels can differ in the size of the orthographic unit (lexical or sublexical) and the contribution of semantics. For example, once the relationship between sublexical orthography and phonology is learnt, it is possible to read new words or pseudowords (e.g. *blig*) that have no semantic associations. Conversely, words with atypical spellings (e.g. *yacht*) can only be read correctly via previously learnt lexico-semantic associations. These observations motivated dual-route theories of reading (Coltheart et al., 1977, Marshall and Newcombe, 1973, Meyer et al., 1974, Morton, 1980), with the sublexical, grapheme to phoneme route being referred to as the ‘indirect’, ‘graphophonological’ or ‘assembled’ route to visual word recognition and the lexico-semantic route referred to ‘direct’ or ‘addressed phonology’. The notion of dissociable routes to phonology is also fundamental to connectionist models that differentially weight the possible links between orthographic and phonological units (Meyer et al., 1974, Plaut et al., 1996, Seidenberg and McClelland, 1989).

Dissociable brain mechanisms underlying lexical and sublexical reading have been indicated by both lesion and functional imaging studies. Lesion data have shown that some patients have more difficulty reading pseudowords than atypically spelt words (e.g. McCarthy & Warrington, 1986) whereas others show the reverse pattern (e.g. Beauvois & Dérouesné, 1979). Likewise, functional imaging studies have reported greater activation for pseudoword than word reading, with the most consistent effect reported in the pars opercularis of the left inferior frontal gyrus (e.g. Brunswick et al., 1999, Fiebach et al., 2002, Fiez et al., 1999, Fiez and Petersen, 1998, Hagoort et al., 1999, Heim et al., 2005, Herbster et al., 1997, Mechelli et al., 2003, Mechelli et al., 2005, Paulesu et al., 2000, Xu et al., 2001). However, it has also been observed that the left pars opercularis is more activated for reading words with irregular than regular spellings, with no significant differences between pseudoword and irregular word reading (Fiez et al., 1999, Mechelli et al., 2005, Nosarti et al., 2010). As irregularly spelled words cannot be read using a phonological assembly strategy, and both types of stimuli are slower to read than regularly spelled words, these authors suggested that activation in the left pars opercularis might simply reflect processing load (Fiez et al., 1999, Mechelli et al., 2005, Nosarti et al., 2010). In contrast, activation in the left dorsal precentral gyrus has been reported to be higher for pseudoword reading than both regular and irregular word reading (Mechelli et al., 2005, Nosarti et al., 2010). The function of the left dorsal precentral gyrus may therefore play a role that is more specific to phonological assembly than that of pars opercularis.

In addition to the influence of processing load, the comparison of pseudoword to word reading is also confounded by inevitable differences in a range of psycholinguistic variables, specifically: frequency and familiarity (by definition, greater for words than pseudowords), orthographic typicality (i.e. bi- and trigram frequencies, which may, paradoxically, be higher in pseudowords than in low-frequency exception words) and associations to meaning (semantics). Not surprisingly, explicit manipulation of each of these word characteristics also leads to modulation of activation within the word reading network (Carreiras et al., 2006, Hauk et al., 2008, Woollams et al., 2011).

In order to tease apart the neural systems involved in assembled and addressed phonology more reliably, Mei et al. (2014) developed an artificial language using an orthography that was unfamiliar to their participants. Native English speakers were taught to read words presented in Korean Hangul characters. Two groups of participants were trained to read words written in this artificial language using either addressed or assembled phonology strategies. Participants who were trained to use an assembled phonology approach activated the left inferior frontal gyrus/precentral gyrus and the left supramarginal gyrus more than those trained to read the words using the addressed phonology strategy. As with the pars opercularis, the left supramarginal gyrus has also been identified in a number of previous studies as involved in being phonological processing (e.g. Price et al., 1997, Sliwinska et al., 2014).

The design of the training study used by Mei et al. (2014) ensured that there were no stimulus differences between the two conditions of interest. However, the use of an artificial language meant that participants read an unfamiliar script. It is unclear whether the reading network recruited during the relatively effortful decoding of an unfamiliar orthographic script can be directly mapped onto the natural reading of a native language in skilled readers.

In the present study, we adopt a novel strategy to investigate phonological assembly within-subjects, using stimuli from their native language. Participants performed a visual lexical decision task in which high-frequency/familiarity concrete nouns (e.g. ‘cake’, ‘mug’, ‘sock’) were contrasted with low-typicality letterstrings (e.g. ‘fpzo’, ‘mwg’, ‘wpwy’). In addition to focusing our planned analyses on contrasts of word *type*, we also manipulated *delivery form*. Letters within the stimulus items were delivered either sequentially or simultaneously. Thus, we argue, phonological assembly is either promoted in the case of sequential stimuli, or not promoted, in the case of simultaneous stimuli. Although this does not guarantee that the participants would rely on assembled more than addressed phonology for the sequentially delivered stimuli, the visual form information that enables whole word recognition is not available for stimuli delivered sequentially until each letter has been identified. This is not the case for the simultaneously delivered stimuli. Therefore, we hypothesised that sequential delivery, relative to the simultaneous delivery, would highlight the brain regions involved in phonology assembly. The non-static delivery of the stimulus makes reading less natural than reading a normal static script. Nonetheless, a significant advantage of this approach is that we were able to use a within-subject design and the same stimulus identities across both delivery forms, thus controlling for individual variability and holding all linguistic properties of the stimuli constant, in the participants’ native language.

We predicted that the sequential delivery of stimuli would result in more activation in parts of the left pars opercularis and supramarginal gyrus that have been associated with using a phonological assembly strategy to read an artificial script (Mei et al., 2014) and the portion of precentral gyrus associated with reading pseudowords more than words with atypical spellings (Mechelli et al., 2005, Nosarti et al., 2010). Furthermore, we tested the hypothesis that these regions would show a lexicality effect (greater activation for words relative to letterstrings), during sequential but not simultaneous delivery of stimuli. Our rationale here was that, during lexical decisions for sequentially delivered items, the demands on visual and phonological memory will be less for letterstrings than words because letterstrings can be rejected as soon as an illegal letter combination is detected, whereas words cannot. Such a finding would indicate that the role of these regions in phonological assembly may be linked to phonological working memory load.

## 2 Methods

### 2.1 Participants

Sixteen participants were scanned for this study. All were right-handed, monolingual, native English speakers. All had normal or corrected-to-normal vision and were without any known neurological or behavioural abnormalities. All participants gave informed, written consent to participate in the study, which was approved by the local Research Ethics Committee. Participants were only included in the fMRI analyses if they made fewer than 25% errors in any one condition and fewer than 15% errors across all conditions. Two participants were excluded on this basis. One further participant was excluded due to excessive head motion in the scanner (greater than a voxel = 3 mm). Data from thirteen participants (six women) were included in the analyses. Their mean age was 30 years (range: 18.8–43.8 years). All tested within the normal range on the block design subtest of the WAIS-R (Wechsler, 1981) (mean percentile: 84.15 [S.D. 13.7]; range 63–98). All were good readers (mean reading age: 18.6 years [S.D. 1.6]; range: 15.5–20.5 years; Vernon-Warden Reading Comprehension Test Revised, 1996) with no reported history of dyslexia.

### 2.2 Experimental design

There were four conditions that orthogonally manipulated lexicality (words vs. letterstrings) and delivery format (sequential vs. simultaneous) resulting in a fully balanced 2 (lexicality) × 2 (delivery format) factorial design (both within subjects factors). Participants made a speeded, forced-choice button-press response to each stimulus item, indicating whether or not the item was a word (visual lexical decision task). For all participants, the right index and middle fingers were used for words and letterstrings, respectively.

### 2.3 Stimuli

There were 256 stimulus identities. Half were English words and half were letterstrings. All word stimuli were highly familiar (>420; range: 420–635; mean: 550; values obtained from MRC database in which familiarity ranges from 43 to 657 with a mean of 488), concrete, one-syllable nouns, either three (*n* = 59) or four (*n* = 69) letters long (e.g. sea; pipe). Stimuli were distributed across four pseudo-randomised presentation lists, which did not differ on length (number of letters), familiarity, concreteness, number and frequency of orthographic neighbours, constrained (positional) bigram frequency, and frequency of word forms sharing constrained bigrams with the stimuli (analogous to the Coltheart N measure – Coltheart et al., 1977). Letterstring stimuli were generated using the MCWord Orthographic Wordform Database (Medler & Binder, 2005), which is based on the CELEX lexical database (Baayen, Piepenbrock, & van H, 1993). Twenty per cent of the letterstrings were pure consonant strings (no vowels); the remainder contained just one vowel. Vowels were included to encourage participants towards a lexico-semantic rather than purely perceptual strategy (i.e. all consonants = ‘not a word’) in performing the lexical decision task. To ensure that the letterstrings were as little word-like as possible, positional bigram frequency for all strings was limited to less than 10 per million (mean: 1.03; range 0–9.88); positional trigram frequency for all strings was zero. Additionally, the frequency of any orthographic neighbours for all letterstrings was less than 10 per million (mean: 1.01; range: 0–9.76). The letterstrings were thus judged unlikely to prompt associations to orthographically similar real words. All letterstrings were either three (*n* = 62) or four (*n* = 66) letters long (e.g. psm; vgux).

**Fig. 1:**
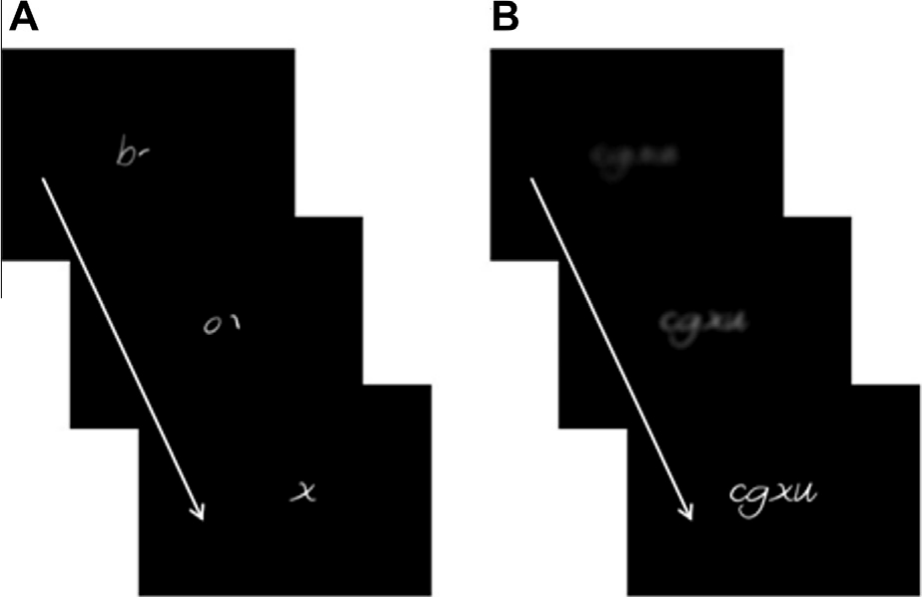
Examples of stimulus delivery formats. Still images presented here are for illustration purposes only; all stimuli were presented as full-motion video clips. (A) *Sequential delivery* – video stills illustrate the sequential unfolding of the English word ‘box’. (B) *Simultaneous delivery* – video stills illustrate the global emergence of the letterstring, ‘cgxu’. Participants performed a lexical decision task on stimuli presented in both delivery formats.

In order to vary delivery format, stimuli were animated using Apple Final Cut Studio (Motion 4) software to create two types of text, which were either: (1) sequentially organised text or (2) simultaneously revealed text. In both cases, stimuli were presented in *Gust* – a sans-serif font, which resembles handwriting. White, lower-case letters against a black background were used. Sequentially delivered stimuli were animated such that individual letters appeared in sequence from left to right, with the strokes comprising each letter appearing in fluid stages. As later letters appeared, preceding letters faded and disappeared. In this way, sequential stimuli never appeared in whole. However, elements of two or three of the letters of each stimulus were visible simultaneously as the item sequentially unfolded. Simultaneously delivered stimuli were at onset both faded and blurred but became progressively brighter and un-blurred until they were clearly visible. The overall shape of the stimuli (perceptual envelope) was perceivable in advance of individual letter identities. Simultaneous stimuli differed most saliently from sequential stimuli in that there was no sequential (internal) ordering of letters but the entire form emerged globally. The timing of presentation of the letters in both conditions was established through extensive piloting. First, the speed of sequential letter presentation at which all pilot participants could achieve 100% accuracy on a lexical decision task was established. The speed of ‘emergence’ of the simultaneous delivery of stimuli was then set so that participants reaction times to lexical decisions (across words and letterstrings combined) were similar across delivery formats. See Fig. 1 for stimulus examples presented as video stills.

### 2.4 Procedure

Each participant completed eight fMRI sessions, of which four are reported here.^2^ Each session lasted 5 min 6 s. A mixed model design was used; words and letterstrings were event-related (pseudo-randomised) within session whereas input form (sequential/simultaneous delivery) was blocked across session. Each session comprised 64 experimental events organised into eight mini-blocks of eight events per block. These mini-blocks were interleaved with null events (fixation cross) of either 12 or 18 s duration. Over the course of the eight fMRI sessions, participants viewed all 256 stimulus identities twice – once in the sessions reported here and once under different task conditions. Repetitions were counterbalanced to reduce the potential for adaptation to stimulus identity (e.g. Grill-Spector, Henson, & Martin, 2006). Stimuli were projected to a screen positioned at the top of the scanner bore; participants viewed the stimuli via a system of mirrors. All stimuli were displayed as 3-s video clips. Stimulus movement started 500 ms after the onset of each clip. For both delivery formats, the durations of 3- and 4-letter stimuli were 1100 ms and 1420 ms, respectively. The fixed SOA was 3 s. Reaction times (RTs) were recorded from the onset of the clip.

### 2.5 MRI acquisition

Anatomical and functional images were acquired from all participants using a Siemens 1.5-T Sonata scanner. Anatomical T1-weighted images were acquired using a 3-D MDEFT (modified driven equilibrium Fourier transform) sequence. One hundred and seventy-six sagittal partitions with an image matrix of 256 × 224 and a final resolution of one mm^3^ were acquired (repetition time (TR): 12.24 ms; echo time (TE): 3.5 ms; inversion time (TI): 530 ms). Structural scans indicated that our participants were free from gross neurological abnormalities and facilitated spatial normalisation of each participant’s data.

Functional T2^∗^-weighted echo-planar images with BOLD contrast comprised 35 axial slices of 2 mm thickness (1 mm gap), resulting in 3 × 3 mm in-plane resolution. One hundred and eight volumes were acquired per session (repetition time (TR): 3.15 s; echo time (TE): 50 ms; flip angle = 90°). TR and stimulus onset asynchrony were mismatched, allowing for distributed sampling of slice acquisition across the experiment (Veltman, Mechelli, Friston, & Price, 2002), which obviates the need for explicit “jittering”. To avoid Nyquist ghost artifacts, a generalised (trajectory-based) reconstruction algorithm was used for data processing. After reconstruction, the first six volumes of each session were discarded to ensure tissue steady-state magnetisation.

### 2.6 Behavioural data analysis

**Fig. 2:**
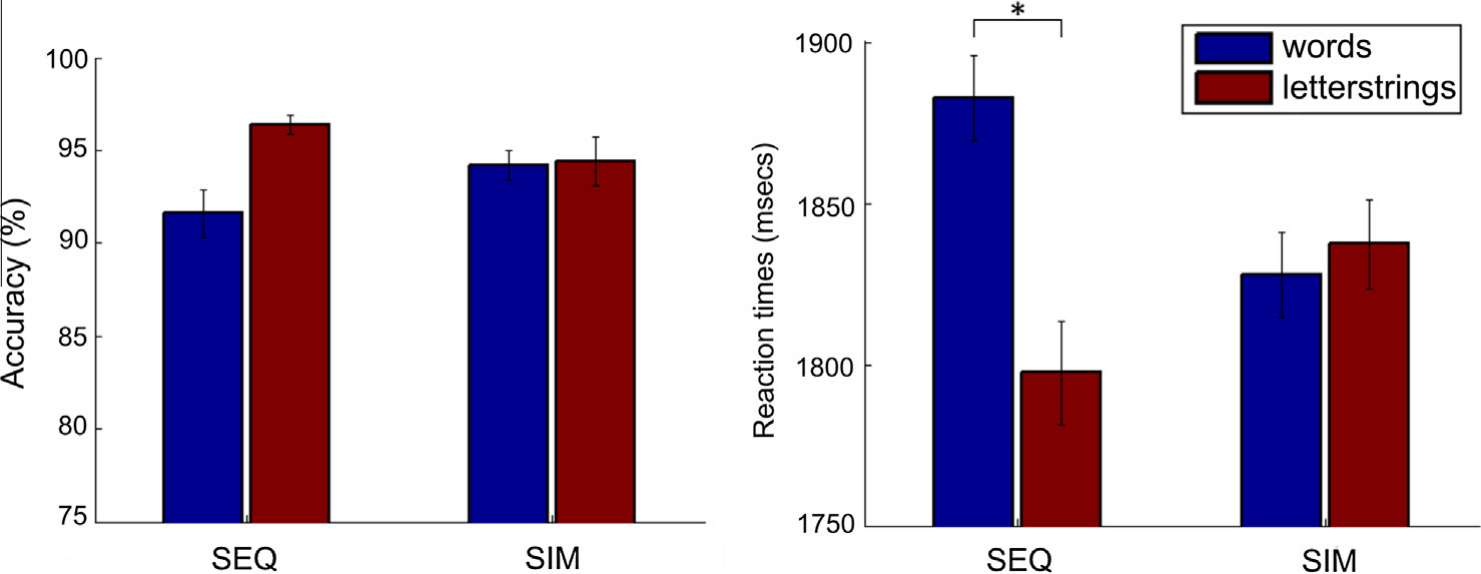
Behavioural data. In the accuracy data there were no main effects of delivery format or lexicality and no interaction (left panel). In the reaction time data (right panel) there was a significant interaction (*F*(1,12) = 22.96, *p* < 0.001). Post-hoc tests indicated that this was due to faster responses to letterstrings than words, only during sequential text delivery. The error bars show corrected standard errors of the mean. SEQ = Sequential delivery; SIM = Simultaneous delivery.

Accuracy and reaction time (RT) data to the lexical decision task (see Fig. 2) were analysed separately using repeated measures ANOVAs with two factors: delivery forms (sequential, simultaneous) and lexicality (words, letterstrings).

#### 2.6.1 fMRI data analysis

Data preprocessing and statistical analyses were performed using SPM8 (Wellcome Trust Centre for Neuroimaging, London UK; http://www.fil.ion.ucl.ac.uk/spm/). Functional images were realigned and unwarped to adjust for the minor distortions in the B0 field due to head movement. These images were then co-registered to the structural image for unified segmentation/normalisation. The realigned and unwarped functional images were spatially normalised to the Montreal Neurological Institute (MNI) 152 space (maintaining the original 3 mm^3^ voxels). Finally the images were spatially smoothed with an isotropic 6 mm FWHM Gaussian kernel.

First-level statistical analyses (individual participants) were based on a least squares regression analysis using the general linear model in each voxel across the whole brain. Low-frequency noise and signal drift were removed from the time series in each voxel with high-pass filtering (1/128 Hz cutoff). Residual temporal autocorrelations were approximated by an AR(1) model and removed. An event-related analysis was used for all trials (no modelling of epoch/block effects); for justification and details, see Mechelli et al. (2003). The first level analyses included the four experimental conditions (sequential words, sequential letterstrings, simultaneous words, simultaneous letterstrings) and additional conditions for incorrect trials. The fixation baseline was implicit and thus not explicitly modelled. For each subject, parameter estimates (i.e. beta images) were assessed with least squares regression analysis. Contrast images (weighted beta images) were computed for each experimental condition (correct trials only) relative to the implicit baseline and entered into the second-level analyses at the group level. These consisted of a 2 × 2 ANOVA with delivery format (sequential, simultaneous) and lexicality (words, letterstrings) both as within-subject factors. For the imaging analyses, we report activation as significant at voxel-level inference of *p* < .05, family wise error corrected for multiple comparisons across the whole brain (*Z* > 4.72) or within the pre-defined ROIs. For completeness we also report significant effects at an uncorrected threshold of *p* < .001 if their extent was greater than 10 voxels across the whole brain.

#### 2.6.2 Regions of interest

Our primary aim was to validate whether sequential delivery of word stimuli increased activation, relative to simultaneous delivery of the same stimuli, in the regions that have previously been associated with “phonological assembly” in studies that have manipulated stimulus type (pseudowords versus words) or strategy to be applied to reading a novel script. Our ROIs for sequential delivery were centred on the coordinates from Mei et al. (2014). The frontal ROI (8 mm radius) was centred at [*x* = −56, *y* = +6, *z* = +24] (labelled as “precentral gyrus/inferior frontal gyrus”) and the posterior ROI was centred at [*x* = −38, *y* = −38, *z* = +40] (labelled as “supramarginal gyrus/superior parietal lobule”). On the mean structural image of the participants in the current study, these coordinates fell within pars opercularis (POp) and supramarginal gyrus (SMG) respectively. The third ROI was centred on the left superior ventral part of precentral gyrus (“superior PCGv”, see Schubotz, Anwander, Knösche, von Cramon, & Tittgemeyer, 2010), which has previously been reported to be more activated for pseudowords than exception words at [*x* = −56, *y* = 0, *z* = +40] (Mechelli et al., 2005).

## 3 Results

### 3.1 Behavioural data

Analysis of the accuracy data indicated no significant main effects of delivery format (*F*(1,12) = .067, *p* = .799) or lexicality (*F*(1,12) = 3.749, *p* = .077) and no significant interaction (*F*(1,12) = 4.070, *p* = .067) (see Fig. 2). These results, combined with a generally high accuracy (>90% for all conditions), suggest that the participants were able to perform the lexical decision task with relative ease despite the novel delivery formats.

Analysis of RT data showed no main effect of delivery (*F*(1,12) = .106, *p* = .751). There was however, a significant main effect of lexicality (*F*(1,12) = 9.832, *p* = .009; responses to letterstrings faster than words) and a lexicality-by-delivery interaction (*F*(1,12) = 22.957, *p* < .001; see Fig. 2). Post-hoc analyses indicated that the interaction was driven by an effect of delivery on words (slower for sequential than simultaneous) with a trend for the reverse on letterstrings. Responses were faster for letterstrings than words during sequential delivery (*t*(12) = 4.835, *p* < .001) with no significant difference between the reaction times for letterstrings and words during simultaneous delivery (*t*(12) = .719, *p* = .486).

### 3.2 fMRI data

**Fig. 3:**
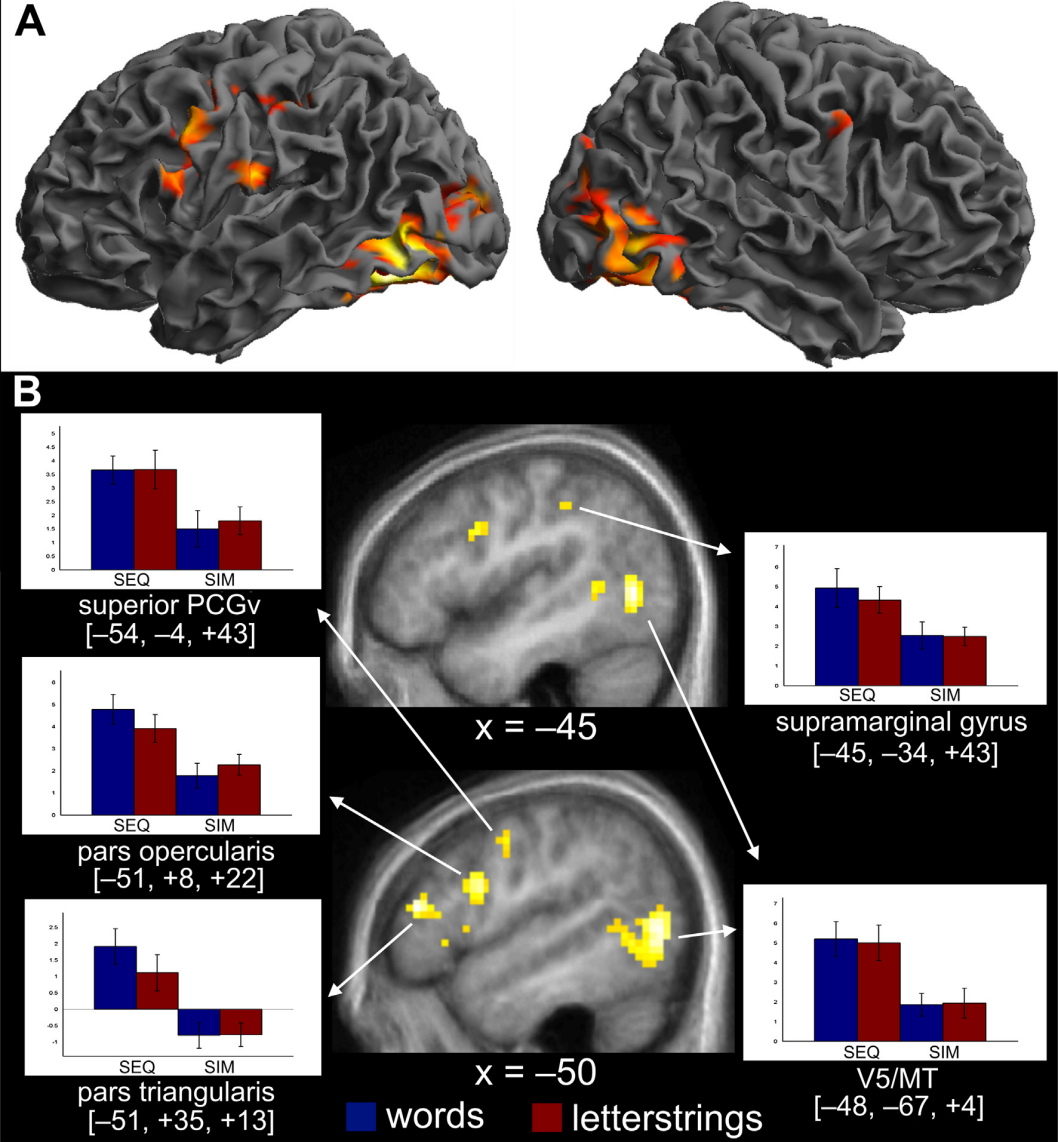
Summary of fMRI results. (A) Main effect of task: areas activated by lexical decision relative to fixation baseline across both sequential and simultaneous delivery. Statistical threshold: *p* < .05 FWE corrected. (B) Bar plots indicating parameter estimates at each region significant either at corrected *p* < .05 level at the whole brain (pars triangularis and V5/MT) or after a small volume correction for the ROI analyses (pars opercularis, ventral precentral gyrus, supramarginal gyrus). Regions where activation was greater for sequential than simultaneous delivery are displayed on the mean structural image from the participants, at the statistical threshold – *p* < .001 uncorrected. SEQ = sequential delivery, SIM = simultaneous delivery.

The main effect of lexical decision, across both delivery formats relative to fixation, indicated activation in a distributed network of regions, in bilateral occipito-temporal, parietal and prefrontal cortices (Table 1 and Fig. 3), consistent with previous studies using visual lexical decision tasks (e.g. Carreiras et al., 2007, Devlin et al., 2006, Fiebach et al., 2007, Hauk et al., 2008, Woollams et al., 2011). These results confirm that the novel delivery formats engaged the typical visual word recognition network.

#### 3.2.1 Sequential versus simultaneous delivery

Two regions were significantly more activated for sequential than simultaneous delivery, after correction for multiple comparisons across the whole brain: a dorsal part of the left pars triangularis (PTr) [*x* = −51, *y* = +35, *z* = +13; *Z* = 4.82] and left V5/MT [*x* = −48, *y* = −67, *z* = +4; *Z* = 4.75]. Our regions of interest were also more activated for sequential than simultaneous delivery, following small volume correction in the left POp [*x* = −51, *y* = +8, *z* = +22; Z = 4.32, *p* = .001), left SMG [*x* = −45, *y* = −34, *z* = +43; *Z* = 3.26, *p* = .039] and left superior PCGv [*x* = −54, *y* = −4, *z* = +43; *Z* = 3.47; *p* = .009]. In no regions was activation greater for simultaneous than sequential delivery after correction for multiple comparisons across the whole brain. See Table 2 for regions identified as differing between delivery formats at a lower statistical threshold (*p* < .001 uncorrected).

#### 3.2.2 Lexicality

There was no significant main effect of lexicality and no lexicality-by-delivery interaction after correction for multiple comparisons across the whole brain. However, as we had predicted an effect of lexicality for sequential – but not simultaneous – delivery, we explored the lexicality effect in both delivery types separately. There was a trend towards greater activation for words than letterstrings during sequential presentation in two of our regions of interest, POp [*x* = −54, *y* = +8, *z* = +19; *Z* = 2.31] and SMG [*x* = −33, *y* = −40, *z* = +40; *Z* = 2.08 and *x* = −45, *y* = −34, *z* = +40; *Z* = 2.00] (identified at *p* < .05 uncorrected. In contrast, no peaks were identified within the PCGv region of interest (see plots in Fig. 3). Regions identified at an uncorrected threshold of *p* < .001 for greater activation for words relative to letterstrings as well as the reverse contrast of letterstrings greater than words are reported in Table 2.

## 4 Discussion

The aim of this study was to test whether three brain regions that have previously been associated with phonological assembly (POp, superior PCGv and SMG) were more strongly activated, during a lexical decision task, when the sublexical components of words and letterstrings are delivered sequentially rather than simultaneously. We reasoned that sequential letter delivery would encourage the use of phonological assembly whilst simultaneous letter delivery would not. Consistent with this hypothesis, we found that, when the demands on phonological assembly increased, activation in our three regions of interest, and also the left pars triangularis (PTr), increased. This finding rules out any possibility that prior association of POp, superior PCGv and SMG with phonological assembly was the consequence of uncontrolled psycholinguistic properties (as when pseudowords are compared to words). Second, our findings also rule out the possibility that activation in these regions can be wholly attributed to general processing load. If this had been the case, observed activation would have been proportional to reaction times. Instead, we found more activation for sequential than simultaneous delivery when response times were both fast (letterstrings) and slow (words; see Fig. 2). Third, our findings demonstrate increased activation in POp, superior PCGv, SMG and PTr when participants were reading in English, their native language, rather than reading an artificial script. The possible role for each of these regions in phonological assembly is discussed below.

### 4.1 A role for the left POp and SMG in phonological memory?

The left POp and SMG are both involved in phonological processing. Furthermore, these regions have also been suggested to co-activate when the ‘controlled’ use of phonological information is required (Gold & Buckner, 2002). In further support of this proposal, the functional connectivity between the left POp and SMG has been shown to be significantly modulated during lexical decision of Japanese Hiragana words, which provide sublexical phonological information, in contrast to words written in Kanji, which do not (Kawabata Duncan et al., 2014).

During sequential stimulus delivery in the current study, each letter had to be held in phonological memory until the participants made a lexical decision. This process is likely to be more demanding for words than letterstrings since letterstrings could be rejected after two or three letters have appeared on the screen, forming an orthotactically illegal letter combination. Indeed, this pattern was reflected in the longer reaction times to sequentially presented words than letterstrings. Although not significant, there was also a trend in both the POp and the SMG towards greater activation for words than letterstrings. The proposal that the left Pop and SMG may be involved in the phonological memory component of phonological assembly, is also supported by studies showing greater activation in this region for stimuli that require more processing demands than words. A previous study of lexical decision reported *less* activation in these regions for words than pseudohomophones (*x* = −42, *y* = +7, *z* = +26 and *x* = −48, *y* = −33, *z* = +46, Twomey, Kawabata Duncan, Price, & Devlin, 2011). Unlike letterstrings used here, pseudohomophones cannot be rejected on the basis of illegal letter combinations and must also be held in phonological memory. This process is likely to be more demanding for pseudohomophones than words, since pseudohomophones sound like a real word but their visual forms are unfamiliar. Future studies using sequential letter presentation could specifically test the hypothesised role of the POp and SMG in phonological memory. For example, by increasing demands on the word processing system by presenting words of longer length or low frequency words.

### 4.2 Superior PCGv: integrating motor speech sequences?

Unlike the left POp and SMG, there was no indication of a trend in left superior PCGv towards increased responses to words relative to letterstrings during sequential delivery.

It has been suggested that the premotor cortex is involved in extracting and predicting a sequential pattern for a goal-oriented action or planning and, importantly, the sequence does not have to be systematic (Schubotz & von Cramon, 2003). This fits well with our results that words (systematic sequences) and letterstrings (non-systematic sequences) activated this region equally during sequential delivery. Furthermore, the response in the premotor cortex corresponds to the appropriate somatotopically organised regions, even though a motor output was not required. The coordinate of our left superior PCGv ROI is close to the area where activation was previously elicited by tongue movement (*x* = −50, *y* = −9, *z* = +36 in Talairach coordinates (TC), Schubotz et al., 2010), covert syllable repetitions (*x* = −48, *y* = −3, *z* = +42, Wildgruber, Ackermann, & Grodd, 2001) and it was found to be the motor cortex’s mouth area in a spatial probability analyses (*x* = −46, *y* = −8, *z* = +40 in TC, Fox et al., 2001). The hypothesis that the left superior PCGv is involved in the integration of sequences of speech motor acts could be tested in future studies by contrasting activation in this region during sequential presentation of words and pronounceable nonwords. Support for the hypothesis would come from greater activation for nonwords than words.

In addition to our regions of interest, two further regions showed a significant difference between sequential and simultaneous word delivery.

### 4.3 Greater activation for sequential than simultaneous delivery: Pars triangularis (PTr)

At the whole brain level we identified greater activation for sequential than simultaneous delivery in the left PTr. This pattern suggests that this region may also be involved in phonological assembly, alongside the regions discussed above. However, the previous literature suggests another, perhaps complementary, interpretation of the greater activation in PTr for sequential than simultaneous presentation. The left PTr has often been reported as activated during lexico-semantic processing (for review see Jobard, Crivello, & Tzourio-Mazoyer, 2003). One of the benefits of the current design is that the same words (and letterstrings), from the participant’s native language, were presented in both delivery formats. Thus there was no difference in semantic content between conditions. However, reaction times suggest that different strategies were used across the two delivery formats. There was an effect of lexicality (words > letterstrings) when stimuli were delivered sequentially, but not simultaneously. Lack of a lexicality effect during simultaneous delivery suggests that participants may not have been accessing semantics during these conditions. As the letterstrings were unpronounceable and easily recognisable as nonwords, it is possible that the lexical decision task was performed on the basis of orthographic familiarity during simultaneous delivery. For the sequentially delivered stimuli however, this information is not available at first and the participants need to identify each letter sequentially. This is likely to lead to activation of words that are the target’s orthographic neighbours and consequently trigger automatic access of their semantic representations. This would be stronger for words than letterstrings since they would yield more orthographic neighbours than letterstrings. The bar plot (Fig. 3B) clearly shows this pattern.

Interestingly, the left PTr was not only greater during sequential delivery, but also deactivated relative to the baseline during simultaneous delivery. Although deactivation is not always reported in the fMRI literature and is not often discussed, it has been demonstrated that the regions that are often deactivated during active tasks and termed as the “default mode network” (Binder et al., 2009, Greicius et al., 2003, Mazoyer et al., 2001, Raichle and Snyder, 2007, Raichle et al., 2001, Shulman et al., 1997) are remarkably similar to those of the general semantic network (Binder et al., 2009). It has been further suggested that deactivation is observed in these regions when task-unrelated thoughts occur during the rest condition, which are semantic in nature. Thus deactivation during the *active* task may reflect the fact that the task places little or no demands on the semantic system (Binder, 2012). Although the left PTr is not in the classic default mode network, deactivation in the present study also fits with this suggestion since it is within the semantic network (Binder & Desai, 2011). We suggest therefore that greater activation in the left PTr during sequential delivery was due to participants accessing more semantic information during sequential than simultaneous word delivery. In support of this conclusion, in the study of Mei et al. (2014) participants using the phonological assembly strategy to read words in an artificial language, with no semantic associations, did not activate this region.

### 4.4 Greater activation for sequential than simultaneous delivery: V5/MT

Finally, we also found greater activation for sequentially delivered than simultaneously delivered stimuli in bilateral V5/MT. This was significant at the whole brain level in the left hemisphere, though the effect in the right hemisphere did not reach the corrected statistical threshold. Our data also showed no activation difference between words and letterstrings in this region (Fig. 3B). Although this pattern is similar to that of the left superior PCGv discussed earlier, activation here is most likely to reflect the physical properties of the stimuli, rather than phonological assembly. It is well-established that this region is sensitive to visual motion (e.g. Born & Bradley, 2005). Although both delivery formats included motion, sequential delivery of letters by necessity led to more apparent, fluid motion than simultaneous delivery. In addition, the increased activation in V5/MT in viewing sequentially delivered letters may relate to the proposed involvement of this area in visual segmentation in addition to simple detection or measurement of visual motion (Born & Bradley, 2005). Indeed, similar activation in V5/MT bilaterally was reported previously (Nakamura et al., 2012) for participants making semantic decisions in response to dynamic, relative to static, orthographic stimuli: similar to watching a pen write, and thus similar to the stimuli in the current study. Importantly however, this activation was observed during perception of both forward and backward writing trajectories, suggesting that it is responsive to sequential motion but not linguistic structure. In line with this, Mei et al. (2014) did not report activation here for their static orthographic stimuli during assembled or addressed phonology.

In conclusion, our data provide clear evidence that the left pars opercularis, the supramarginal gyrus and the precentral gyrus all play an important role in phonological assembly during reading. In contrast to previous studies, our conclusion is not confounded by differences in psycholinguistic factors between stimuli. Furthermore participants were tested on a familiar orthography. We observed remarkably similar patterns of activation to previous lexical decision studies using static stimuli, but different patterns of activation for stimulus delivery formats. We also found greater activation for sequential than simultaneous delivery in the left pars triangularis. On the basis of findings in the previous literature, we propose the involvement of this region in reading words with sequential letter presentation is most likely related to semantic activation of orthographic neighbours of the target word. The trend in the data towards an effect of lexicality in some regions but not others, raises the interesting possibility that manipulation of letter presentation could be used to identify the different roles played by these regions in the phonological assembly process. Future studies contrasting sequential and simultaneous letter presentation are needed to investigate this possibility further by, for example, increasing demands on the word processing system by manipulating word length or using low frequency words. Our data suggest that sequential delivery of orthographic stimuli is a useful tool to further explore the use of sublexical phonological processing in visual word recognition, both in skilled and less skilled readers.

## Figures and Tables

**Table 1:** Regions identified in the main effect of lexical decision task (words and letterstrings combined) across both delivery formats, relative to fixation at *p* < .05 FWE corrected.

Region		Mean peak coordinate			*Z*-score
		*x*	*y*	*z*	
*Occipital*					
L	Posterior vOT	−45	−64	−14	7.72
R	Posterior vOT	+45	−61	−11	7.66
L	Anterior vOT	−39	−49	−17	7.01
L	Lingual gyrus	−18	−82	−17	7.62
L	Cuneus	−3	−76	+10	5.12

*Frontal*					
L	Precentral gyrus	−42	+2	+31	7.81
L	Precentral gyrus	−51	+2	+43	6.42
R	Precentral gyrus	+42	+5	+31	5.84
L	Pre-SMA	−3	+8	+52	7.65
R	Pre-SMA	+3	+17	+43	6.69
L	Frontal operculum	−42	−4	+10	4.86

*Parietal*					
L	Parietal operculum	−60	−19	+19	7.05
L	Intraparietal sulcus	−27	−73	+28	7.01
R	Intraparietal sulcus	+30	−64	+37	6.58
L	Supramarginal gyrus	−51	−31	+46	6.02

*Subcortical*					
L	Thalamus	−12	−16	+7	5.52
L	Putamen	−24	−1	+4	4.99
R	Cerebellum	+27	−49	−23	7.71
R	Cerebellum	+6	−58	−14	6.84

Abbreviations: vOT = ventral occipitotemporal cortex, SMA = supplementary motor area.

**Table 2:** Peak coordinates identified for each contrast at *p* < .001 uncorrected (*k* > 10). See text for those significant at corrected level.

Region		Mean peak coordinate			*Z*-score
		*x*	*y*	*z*	
*Sequential > simultaneous*					
R	Pars triangularis	−48	+38	+10	4.41
R	Cerebellum	+18	−70	−32	4.57
L	Pars opercularis (dorsal)	−51	+8	+22	4.32
L	Intraparietal sulcus	−27	−76	+31	4.04
R	Middle frontal gyrus	+39	+53	+4	3.98
L	Pars opercularis (ventral)	−57	+17	+7	3.87
R	MT/V5	+54	−64	+7	3.85
R	Pars opercularis (dorsal)	+45	+8	+16	3.63
L	Insula	−39	−4	+4	3.61
L	Precentral gyrus	−54	+4	+43	3.47

*Simultaneous > sequential*					
R	Fusiform gyrus	+31	−76	−11	4.64
+27	−58	−11	3.84
L	Fusiform gyrus	−21	−79	−11	3.35

*Words > letterstrings*					
L	Frontal operculum	−36	+20	+4	3.92

*Letterstrings > words*					
R	Superior temporal sulcus	+39	−43	−10	4.26
R	Middle frontal gyrus	+45	+50	+1	4.25
R	Frontomarginal sulcus	+24	+50	−8	4.06
R	Superior frontal sulcus	+18	+38	+34	3.97
R	Frontomarginal gyrus	+24	+53	+10	3.86

## Footnotes

2 Unreported sessions comprised fingerspelled (British two-handed manual alphabet) stimuli and an additional task (feature detection).
